# Evaluation of an extracorporeal ozone-based bactericide system for the treatment of *Escherichia coli* sepsis

**DOI:** 10.1186/s40635-022-00443-w

**Published:** 2022-04-25

**Authors:** Paul Skorup, Anette Fransson, Jenny Gustavsson, Johan Sjöholm, Henrik Rundgren, Volkan Özenci, Alicia Y. W. Wong, Tomas Karlsson, Christer Svensén, Mattias Günther

**Affiliations:** 1 Section of Infectious Diseases, Department of Medical Sciences, Uppsala University, Uppsala, Sweden; 2 Section for Experimental Traumatology, Department of Neuroscience, Karolinska Institutet, Biomedicum – 8B, 171 77 Stockholm, Sweden; 3 Sangair AB, Stockholm, Sweden; 4 Division of Clinical Microbiology, Department of Laboratory Medicine, Karolinska Institutet, Huddinge, Sweden; 5 Department of Clinical Microbiology, Karolinska University Hospital, Huddinge, Sweden; 6 Department of Clinical Science at Education Södersjukhuset, Unit of Anesthesiology and Intensive Care, Karolinska Institutet, Stockholm, Sweden

**Keywords:** Porcine sepsis model, Ozone, *E. coli*, Antibiotic resistance, Intensive care

## Abstract

**Background:**

Sepsis is associated with substantial mortality rates. Antibiotic treatment is crucial, but global antibiotic resistance is now classified as one of the top ten global public health risks facing humanity. Ozone (O_3_) is an inorganic molecule with no evident function in the body. We investigated the bactericide properties of ozone, using a novel system of extracorporeal ozone blood treatment. We hypothesized that ozone would decrease the concentration of viable Escherichia coli (*E. coli*) in human whole blood and that the system would be technically feasible and physiologically tolerable in a clinically relevant model of *E. coli* sepsis in swine.

**Methods:**

The *E. coli* strain B09-11822, a clinical isolate from a patient with septic shock was used. The in vitro study treated *E. coli* infected human whole blood (*n* = 6) with ozone. The in vivo 3.5-h sepsis model randomized swine to *E. coli* infusion and ozone treatment (*n* = 5) or *E. coli* infusion and no ozone treatment (*n* = 5). Live *E. coli*, 5 × 10^7^ colony-forming units (CFU/mL) was infused in a peripheral vein. Ozone treatment was initiated with a duration of 30 min after 1.5 h.

**Results:**

The single pass in vitro treatment decreased *E. coli* by 27%, mean 1941 to 1422 CFU/mL, mean of differences − 519.0 (95% CI − 955.0 to − 82.98, *P* = 0.0281). pO_2_ increased (95% CI 31.35 to 48.80, *P* = 0.0007), pCO_2_ decreased (95% CI − 3.203 to − 1.134, *P* = 0.0069), oxyhemoglobin increased (95% CI 1.010 to 3.669, *P* = 0.0113). Methemoglobin was not affected. In the sepsis model, 9/10 swine survived. One swine randomized to ozone treatment died from septic shock before initiation of the treatment. Circulatory, respiratory, and metabolic parameters were not affected by the ozone treatment. *E. coli* in arterial blood, in organs and in aerobic and anaerobic blood cultures did not differ. Hemoglobin, leucocytes, and methemoglobin were not affected by the treatment.

**Conclusions:**

Ozone decreased the concentration of viable *E. coli* in human whole blood. The system was technically feasible and physiologically tolerable in porcine sepsis/septic shock and should be considered for further studies towards clinical applications.

## Introduction

Bacterial infections may progress to sepsis and septic shock, which are conditions associated with substantial mortality rates [1, 2]. Antibiotic treatment is crucial for severe infections, but global antibiotic resistance is increasing and is now classified as one of the top ten global public health risks facing humanity by the World Health Organization [3, 4]. The development of new antibacterial agents is insufficient and alternate bactericide treatments are desirable [5]. Ozone, or trioxygen, is an inorganic molecule with the chemical formula O_3_. Ozone is formed from dioxygen (O_2_) by the action of ultraviolet (UV) light and electrical discharges within the Earth's atmosphere [6]. Except for the presence of an ozone-like compound in atherosclerotic plaques, there is no evidence of a physiological function in the human body [7], but some experimental and clinical evidence suggest a medical use of ozone [6, 8]. Ozone generates reactive oxygen species (O_2_^−^, OH, H_2_O_2_, NO and HOCl), also produced by granulocytes and macrophages during an infectious process which may kill bacteria by oxidative stress surpassing redox defenses of the bacteria [9]. Ozonated water destroys bacterial biofilms in vitro [10], and it is possible that ozone could also kill bacteria in blood. A study of *Staphylococcus aureus*, methicillin-resistant Staphylococcus Aureus (MRSA) and Pseudomonas aeruginosa suspended in culture media concluded that ozone did not kill bacteria when 5% human plasma was present, while bacteria suspended in protein-free saline were killed at high ozone concentrations [11]. Ozone also reduces concentrations of viable bacteria in milk, which may be applicable for the dairy industry [12]. It is possible that ozone kills bacteria in blood, and since *E. coli* is the most common Gram-negative pathogen causing sepsis and septic shock, we created an extracorporeal model to test this [13]. In this proof-of-concept study we hypothesized that ozone would decrease the concentration of viable *E. coli* in human whole blood and that the system would be technically feasible and physiologically tolerable in a clinically relevant model of *E. coli* sepsis in swine.

## Methods

The in vitro human whole blood experiments were approved by the Swedish Ethical Review Authority (approval no: 2021-01181). The animal experiments were approved by the Animal Ethics Committee in Linköping, Sweden (approval no: 3264, 12578-2020/17635-2020). The swine were handled in accordance with the Guide for the Care and Use of Laboratory Animals. The surgery was performed under general anesthesia and efforts were made to minimize suffering. Animals had water ad libitum at all times, and food until 12 h before the experiment.

### Organism

The *E. coli* strain B09-11822 was a clinical isolate obtained from a patient with bloodstream infection and septic shock. This strain was encapsulated and serum resistant (analyzed to be serotype O-rough:K1:H7 at Statens Seruminstitut, Copenhagen, Denmark). In vitro pilot studies demonstrated continued growth in both serum, swine whole blood and human whole blood (data not shown) and the strain was employed successfully in a porcine model of severe sepsis [14]. The bacteria were harvested, grown to a logarithmic growth phase, and prepared as previously described.

### Extracorporeal blood ozonation

**Fig. 1:**
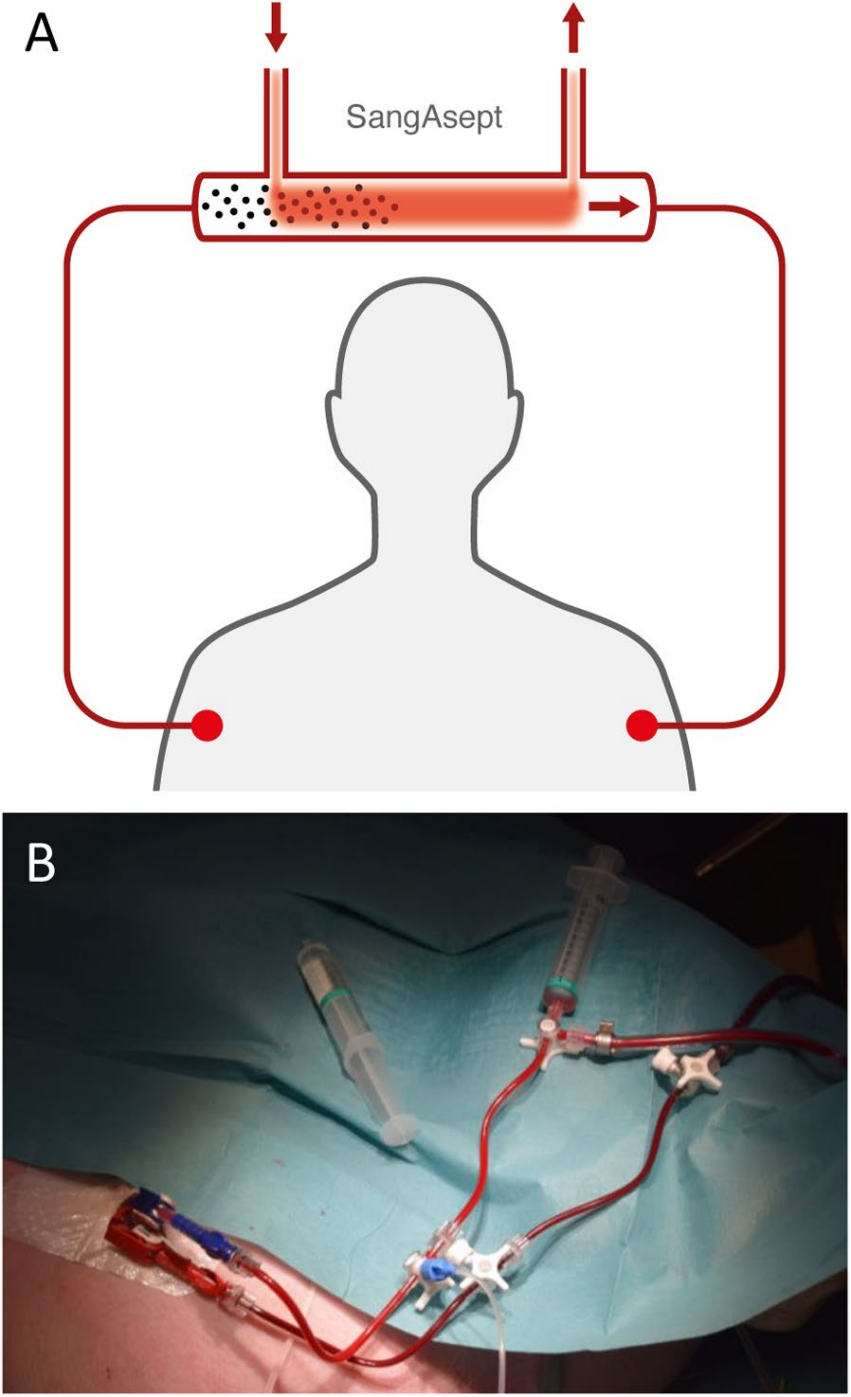
**A** Principal working mechanism of the ozone system. Venous blood is collected from the patient, decreased in temperature, mixed with ozone, reheated, and returned to the patient. **B** Photo of the central venous line (dialysis catheter) in the right femoral vein, showing dark red venous blood transported to the ozone system from the red connector, and light red, oxygenated blood returned from the oxygenator to the blue connector

The SangAsept blood ozonation prototype (patent no WO2016/043649) treated blood with ozone. Briefly, blood was pumped from the venous system of a swine, or from a container with human whole blood (mean blood flow 49–51 mL/min) to a section, where the blood was cooled (mean cooling of blood to 7–9 °C), and then treated with a gaseous 90% O_2_/10% O_3_ mixture. The ozone concentration was < 100 g/m^3^ and the gas flow was < 100 mL/min. The solubility of ozone increases with colder temperatures [15]. The blood was then warmed to a mean of 25–29 °C before being returned the blood to the swine, or for the in vitro studies, to a collecting container (Fig. 1a, b).

### In vitro experiments in human whole blood

Bags of human whole blood were acquired from the blood bank in Stockholm. One bag weighted approximately 200–450 g. 1–3 bags from the same blood group were pooled in a sterile container. *E. coli* 3.4 × 10^8^ CFU/mL diluted in saline were added therein for an in vitro bacterial concentration of 3–5 × 10^4^ CFU/mL (4.6 ± 0.25 log_10_ CFU), 20 min before start. The ozonation prototype was connected by a hose to the container, and the blood was treated one time through the system (*n* = 6). Blood samples were collected during ozone treatment, both upstream and downstream from the machine, for quantitative bacteria analyses and blood gases.

### In vivo experiments in swine

**Fig. 2:**
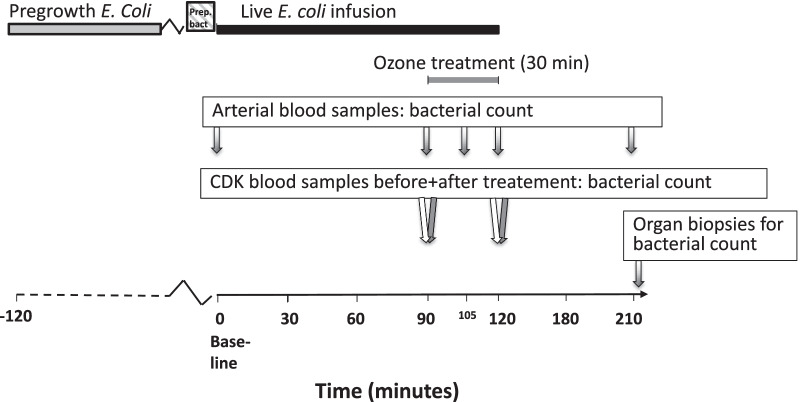
Experimental setup of the swine sepsis model

10 landrace male swine with a mean weight of 62 (range 56–68) kg were used. The experimental setup is described in Fig. 2. Pre-medication consisted of 150 mg tiletamine/zolazepam (Zoletil 100 Vet) and 6 mg medetomidine (Cepetor) after which anesthesia was induced with fentanyl 2.5 µg/kg and pentobarbitalnatrium 6 mg/kg. Endotracheal intubation was performed with a custom-made Miller-type laryngoscope using a standard cuffed size 8 tube. Throughout the study, perioperative hypnosis was maintained with ketamine 25 mg/kg/h and midazolam 0.0485 mg/kg/h, and analgesia with fentanyl 3.5 μg/kg/h. The animals were ventilated with a Hamilton C2 (Hamilton Medical, Geneva, Switzerland) using pressure control with initial settings PEEP 4, PIP 15 cm H_2_0, respiratory rate 15/min and FiO_2_ always remained at 21%. Settings were adjusted to achieve normoventilation (PaCO_2_ 4.9–5.7 kPa). A 7.5 F, 110 cm pulmonary artery catheter (Edwards Lifescience, Irvine, California USA) was cannulated in the right internal jugular vein via cut-down for monitoring of central venous pressure (CVP), cardiac output (CO), pulmonary artery pressure (PAP), mixed venous saturation (SvO_2_) pulmonary arterial wedge pressure (PAWP) and core temperature (Vigilance II-monitor, Edwards Lifescience). Arterial blood gases (Hb, MetHb, pH, PaCO_2_, PaO_2_, Na^+^, K^+^, Ca^++^, glucose, lactate, Hct, SaO_2_, pO_2_, pCO_2_, base excess) were collected on baseline and then repeatedly throughout the experiment (GEM Premier 4000, Instrumentation Laboratories, USA, and Biopac Systems, USA). A 13.5 F, 15 cm double lumen dialysis catheter (medCOMP, USA) was cannulated in the right femoral vein for the ozone system. Perioperative monitoring by continuous electrocardiograms, and urine output were also performed. After an initial fluid bolus of 500 mL Ringer’s Acetate at induction of anesthesia to correct for individual differences in preoperative fluid balance, the infusion rate was 3 mL/kg/h during anesthesia. 100 mL boluses were given if mean arterial pressure (MAP) < 35 mmHg. No blood autotransfusion was performed. To simulate an intensive care setting, animals were treated in accordance with a protocol to maintain vital parameters within preset limits, as previously described [14]. Briefly, arterial partial pressure of oxygen (PaO_2_) was maintained above 10 kPa, mean arterial pressure (MAP) at ≥ 60 mmHg and cardiac index (CI) at ≥ 2 Lxmin^−1^ xm^−2^.

After preparation, animals were randomized by random numbers to *E. coli* infusion and ozone treatment (*n* = 5) or *E. coli* infusion and no ozone treatment (*n* = 5). Live *E. coli*, 1.2 × 10^8^ colony-forming units (CFU/mL) in 20 mL normal saline was infused at baseline in a peripheral vein catheter at a constant infusion rate of 7.1 mL/h for 2 h. To prevent blood from clotting in the extracorporeal ozone system, 4000 E heparin (66.6 E/kg) anticoagulation was administered intravenously at 15 min before start of the system, and then by a continuous infusion of 2000 E/h (33.3 E/kg/h) during the 30 min ozone treatment. Blood samples were collected at baseline and at specific timepoints during the *E. coli* infusion for bacterial cultures. The number of bacteria in the infusate was monitored, with an accepted range 0.9–1.6 × 10^8^ CFU/mL (8.1 ± 0.25 log_10_ CFU). The experiment was terminated 90 min after the bacteria infusion had ended. The animals were euthanized by 40 mL pentobarbitalsodium (Alfatal Vet 100 mg/mL), and post-mortem examinations were performed. Organ samples of 1–2 g were obtained using a sterile technique within 20 min from euthanasia, from standardized locations in the kidney, liver, lung, and spleen.

### Bacteria quantification from blood and organ samples

Bacteremia was determined by blood cultures of 10 mL blood each in two blood culture bottles, one BacT/Alert-FA Plus and one BacT/Alert-FN Plus blood culture bottle (bioMérieux, Marcy-l'Étoile, France). All blood culture bottles were incubated in the automated BacT/ALERT 3D blood culture system (bioMérieux, Marcy-l'Étoile, France) until positivity, or for a maximum of 5 days. Time to detection (TTD) for bottles in the study were also noted. The blood cultures were indexed between 0 and 100 according to bacterial occurrence and TTD. Bacterial quantification was also performed by quantitative bacterial determination of blood and organs on cysteine lactose electrolyte deficient (CLED) agar plates (Becton Dickinsson). 0.1 mL blood was aliquoted on agar plates in triplicates and cultured at 37 °C overnight and bacteria were quantified with viable count technique. CDK blood samples 0.1 mL were collected upstream and downstream from the extracorporeal ozone system before initiation of the treatment and after 15 min of treatment had ended and aliquoted on CLED plates in duplicates for bacteria quantification (CFU per mL). The organ samples were weighed, minced, placed in sterile mortars, and homogenized in 3 mL saline and 0.2 mL of that mixture aliquoted on CLED plates in triplicates for bacteria quantification, CFU per gram.

### Statistical analyses

Statistical analyses were performed using GraphPad Prism version 9.2.0 for Windows (GraphPad Software, La Jolla, Ca). The primary outcome was the concentration of viable *E. coli* in human whole blood. *P* < 0.05 was considered statistically significant. Error bars represent the standard deviation. This study was a primary feasibility study which is why a power calculation was not used. For in vitro* E. coli* in arterial blood, pO_2_, pCO_2_, MetHb and O_2_Hb, two-tailed, paired *t* tests were used. All data were normally distributed (Shapiro–Wilk test). In vivo temporal data sets, and *E. coli* in arterial blood and in organs and aerobic and anaerobic blood cultures in vivo were analyzed using a mixed-effects model (REML), fixed effects (type III) with Šídák’s multiple comparisons test.

## Results

**Fig. 3:**
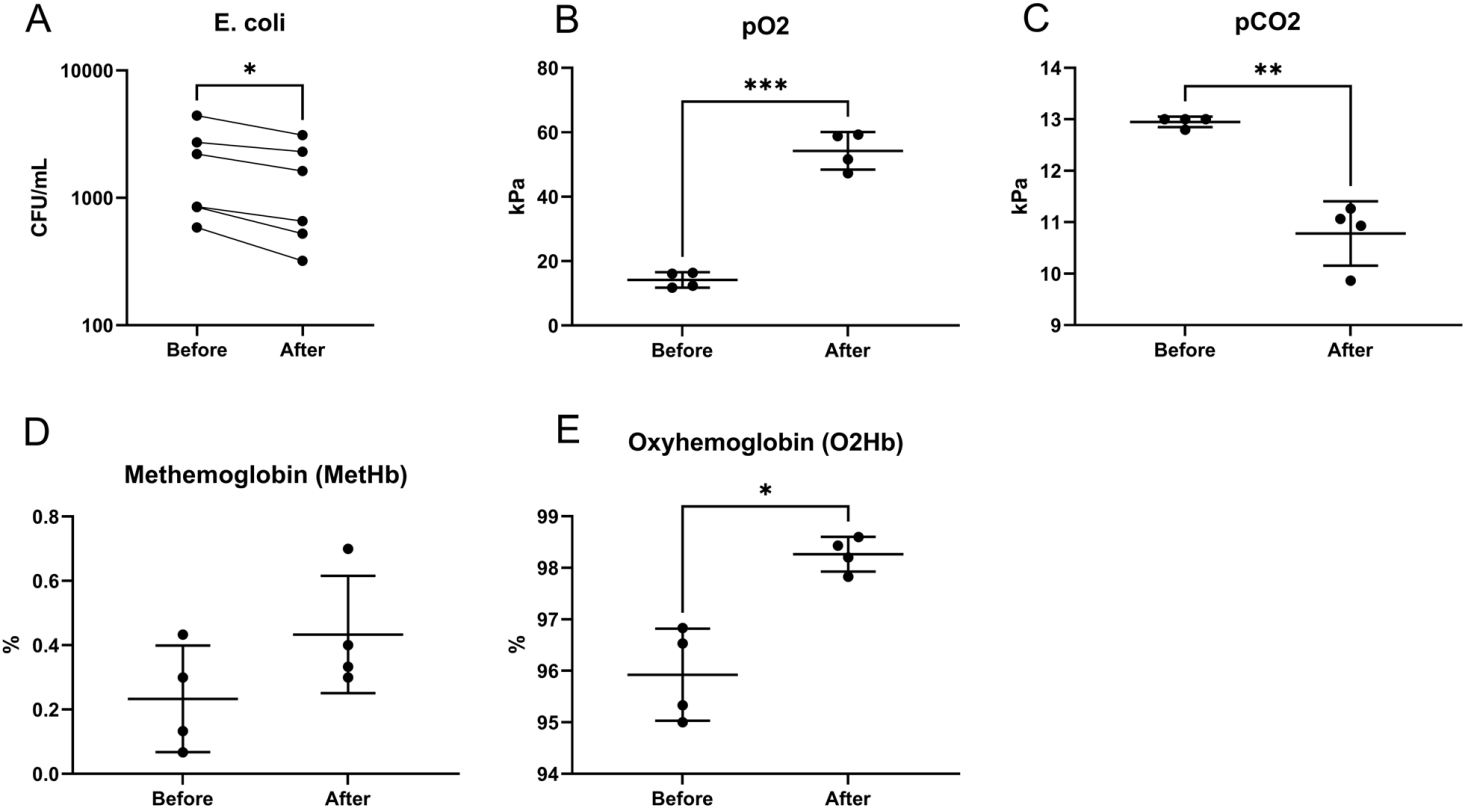
In vitro human whole blood in a single pass ozonation. **A** Ozonation decreased *E. coli* by 27% (*p* < 0.05). **B** pO_2_ increased (*p* < 0.005). **C** pCO_2_ decreased (*p* < 0.01). **D** Methemoglobin, a marker of oxidative stress, was not affected. **E** Oxyhemoglobin increased. *p < 0.05, **p < 0.01, ***p < 0.005

**Fig. 4:**
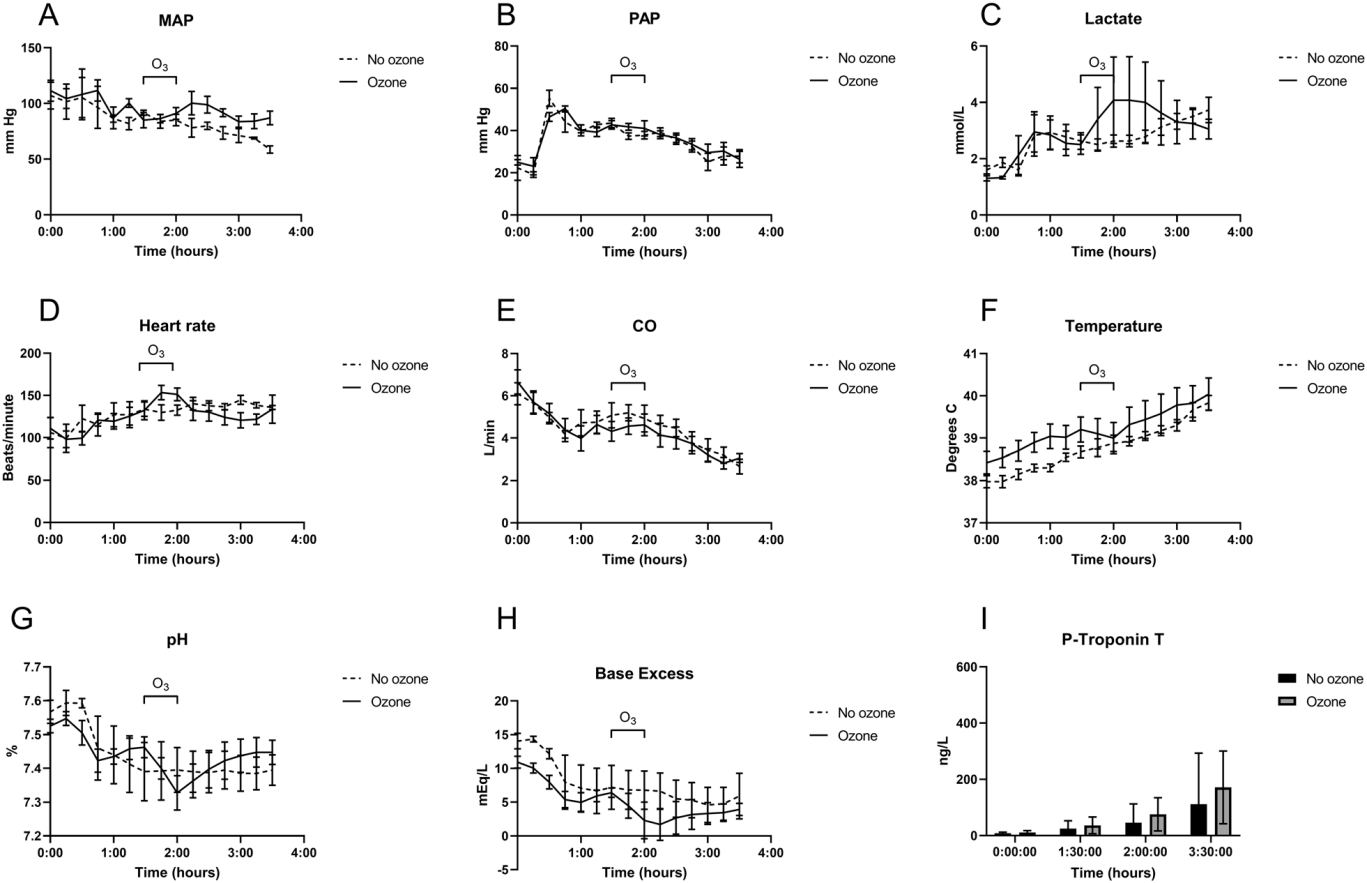
In vivo swine physiological response to *E. coli* sepsis and ozone treatment. **A**–**I** MAP, PAP, lactate, heart rate, CO, temperature, pH, Base Excess and Troponin I were affected by sepsis but not affected by ozone treatment

**Fig. 5:**
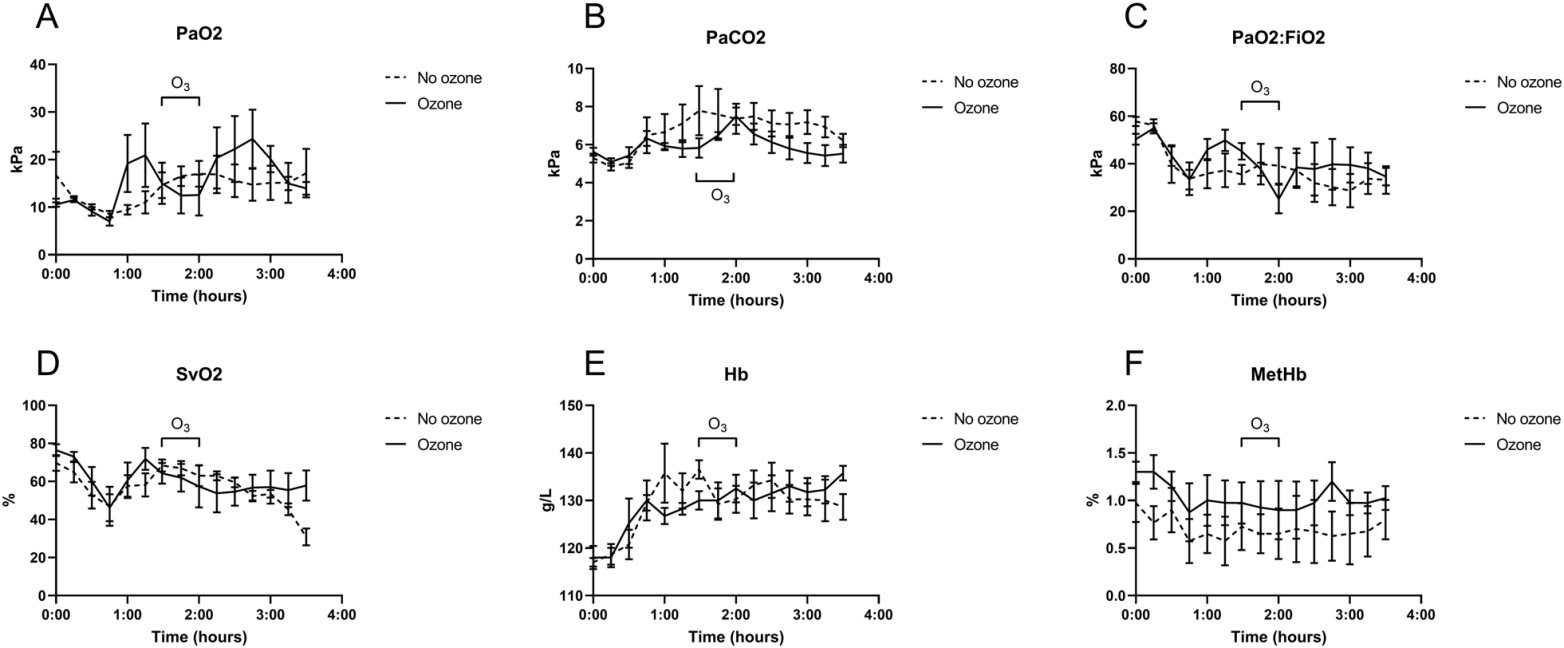
In vivo swine physiological response to *E. coli* sepsis and ozone treatment. **A**–**F** PaO_2_, PaCO_2_, PaO_2_:FiO_2_, SvO_2_, Hb, MetHb were affected by sepsis but not affected by ozone treatment

**Fig. 6:**
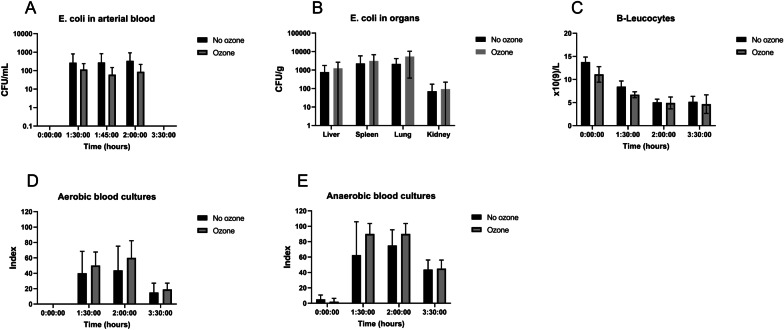
Microbiology of in vivo swine *E. coli* sepsis. **A**–**E** *E. coli* in arterial blood, in organs, leucocytes, aerobic, and anaerobic blood cultures were not affected by ozone treatment. Leucocytes decreased as a result of sepsis

In vitro experiments were performed in human whole blood. A single pass treatment decreased *E. coli* by 27%, mean 1941 to 1422 CFU/mL, mean of differences − 519.0 (95% CI − 955.0 to − 82.98, *P* = 0.0281) (Fig. 3A). pO_2_ increased by 283%, mean 14.18 to 54.26, mean of differences 40.08 (95% CI 31.35 to 48.80, *P* = 0.0007) (Fig. 3B). pCO_2_ decreased by 17%, Mean 12.95 to 10.78 mean of differences − 2.169 (95% CI − 3.203 to − 1.134, *P* = 0.0069) (Fig. 3C). Methemoglobin did not differ (Fig. 3D). Oxyhemoglobin increased by 2.34 percentage points, mean 95.92 to 98.26, mean of differences 2.340, *P* = 0.0113 (95% CI 1.010 to 3.669, *P* = 0.0113) (Fig. 3E). In vivo experiments were performed in swine. Nine swine survived the sepsis model and one swine randomized to ozone treatment died from septic shock before initiation of the treatment. The 10% mortality was within the expected mortality rate of the model. Circulatory and metabolic effects from the sepsis induction and the 30-min ozone treatment were compared. MAP, PAP, lactate, heart rate, CO, temperature, pH, Base Excess and Troponin T did not differ between groups (Fig. 4A–I). Respiratory effects from the sepsis induction and the 30-min ozone treatment were then compared. PaO_2_, PaCO_2_, PaO_2_:FiO_2_ and SvO_2_, did not differ between groups (Fig. 5A–D). Hb and MetHb did not differ between groups (Fig. 5E, F). The in vivo microbiological effects were then assessed. *E. coli* quantification in arterial blood and organs, and B-leucocytes did not differ (Fig. 6A–C). Nor were differences in aerobic or anaerobic blood cultures detected (Fig. 6D–E).

## Discussion

In this study we showed indications that extracorporeal ozonation decreased the concentration of viable *E. coli* in human in vitro whole blood, and that the system was technically feasible and physiologically tolerable in a porcine model of severe *E. coli* sepsis/septic shock.

First, we investigated whether ozone could decrease the *E. coli* concentration in human whole blood. *E. coli* is the most common pathogen causing bacteremia, and a major cause of morbidity and mortality in hospitals, particularly in intensive care units [16–18]. We utilized a strain collected from a Swedish patient with septic shock in a Swedish university hospital, ensuring clinical relevance of the pathogen [14]. In comparison, laboratory strains of *E. coli* may be weak and nonvirulent [19, 20]. Ozone decreased *E. coli* with a rate of 0.69 by a single pass through the ozonation chamber, indicating that ozone was able to decrease the number of viable bacteria in blood. The ozonation also increased pO_2_, increased oxyhemoglobin, and interestingly, decreased pCO_2_. Improved oxygen content in the blood may be favorable in a patient with septic shock and subsequent oxygen debt.

Second, we investigated the ozonation system in a sepsis model in swine [14]. The intention was primarily to evaluate if the system was practically and physiologically tolerable in severe sepsis, and secondarily to evaluate if the system could decrease levels of bacteria. To simulate a clinical situation when immediate and appropriate antibacterial treatment is essential, such as sepsis in an intensive care unit, a model of sepsis/septic shock was deemed accurate. The animals were infused with the same *E. coli* strain as in the in vivo experiments, and subsequently developed severe sepsis with signs of circulatory shock. One out of ten animals died during bacteremia despite optimal ICU resuscitation, which we considered relevant for the model severity and reflected the high mortality in these infections [21]. The modus of in vivo bacteria infusion and study length was based on our previous experience [14, 22] and chosen to establish measurable bacteremia and localized measurable bacteria in immuno-active solid organs. The timing of ozone treatment at 105 min were chosen, because most bacteria had then been infused, to measure an antibacterial effect. After initiation of severe sepsis, the ozone system was connected to a standard dialysis catheter in the femoral vein. The system was manageable in an intensive care setting and caused no signs of physiological adverse effects in circulation, pulmonary artery pressure, or temperature. Blood gases were not affected by the system. Troponin T increased but was not affected by the system. Ventilation, evaluated by pO_2_, pCO_2_ and PaO_2_:FiO_2_, were not affected by the system. We concluded that the ozonation system was tolerable for the 30-min running time in a model of a physiologically compromised sepsis patient. Moreover, the number of blood cells were not affected; Hb increased during the experiment and the leucocyte count decreased because of sepsis. No difference was detected between groups. It is still possible that leucocytes were affected in antibacterial activity. Neutrophil phagocytic activity increases during ozone administration [23, 24], which may be favorable in sepsis, and should be further investigated. It is also possible that the general decrease of leucocytes by sepsis prevented from detecting effects on leukocyte count by ozone alone.

We then evaluated the antibacterial effect. No difference was detected in bacteria presence during the treatment or at the end of the experiments, and no differences were detected in organ cultures or in anaerobic or aerobic blood cultures. Swine are inherently competent in spontaneous clearance of *E. coli* in blood, which is why bacteria was continuously infused during the experiment, as earlier described [14]. For technical reasons we limited the ozone administration to 30 min. It is likely that ozone concentration, blood flow through the ozonation chamber and application time of the system require optimization to achieve a similar bactericide effect as in the in vitro studies. It is also possible that bacterial levels, type of bacteria and blood components, such as albumin levels affect the performance. Such optimizations were beyond the scope of this initial investigation but will be the focus of future studies, which should also include other common pathogens in sepsis, including streptococci and staphylococcus aureus. Due to the pilot-nature of this in vivo study, no titration of dose and time of exposure were performed but remains to be investigated following the established antibacterial effect in vitro.

The oxidative stress levels induced by ozone and the ability to kill bacteria should be weighed against potential harmful effects to the blood, by dosing ozone in levels not exceeding what the antioxidative system in the blood cells may neutralize [6, 25]. Blood contains 55% plasma and 45% cells, primarily erythrocytes. Erythrocytes contain potent antioxidant systems including growth stimulating hormone, thioredoxin, catalase, GSH-Rd, GSH-Px, GSH-Tr, and SOD, all of which are capable of neutralizing oxidants, such as ·OH, H_2_O_2_, OCl^−^ and ONOO^−^ [6, 26]. Organs and cells in the body have different antioxidant capabilities. For example, the alveolar surface in the lung is protected by a small volume of lining fluid which, by having a small antioxidant content, cannot neutralize the oxidant activity of ozone [6, 26]. A comparative analysis between the lungs and blood has clarified the possibility of using ozone as a therapeutic agent provided that dosages are not overwhelming the antioxidant capacity of blood [6, 27]. The dosing of ozone may also be dependent on the number of bacteria and blood cells in the blood. Ozone does not follow the classical Henry’s law in terms of linear solubility of oxygen with pressure, because it reacts immediately with bacteria, ions and biomolecules in the blood. Ozone is tenfold more soluble than oxygen, as ozone dissolves in plasma and instantaneously reacts with hydrophilic antioxidants [26, 28]. Therefore, the exact dosing in comparison to bacterial levels should be the focus of future studies. An initial indication of oxidative stress in the blood was provided by methemoglobin, a form of oxidized hemoglobin (Hb) normally maintained as a very small proportion of total hemoglobin (< 1%). Clinically significant methemoglobinemia can occur due to acquired exposure to oxidizing medications [29]. Methemoglobin did not increase in vitro or in vivo. It is, therefore, possible that the ozone dose did not lead to excessive oxidative damage, although future studies should investigate oxidative stress comprehensively, including markers of DNA damage and lipid peroxidation [28, 30, 31]. Moreover, we observed a non-significant rise in lactate, which may increase mortality in sepsis. While this rise was an indication of an increase, this should be further analyzed in subsequent studies with longer survival times.

There are some more limitations to be discussed. First, the in vitro study had no control arm without ozone treatment. However, the *E. coli* strain was well described to thrive and grow in blood in vitro [14]. Second, while the immune system and organ functions of swine resemble those of humans [32, 33], and the anatomy and physiology of the swine are similar [34], the *E. coli* clearance may be increased compared to humans [32]. While this limits the transferability of the model, swine are still considered a valid species for sepsis models [35]. Third, animals receiving no *E. coli* infusion were not included in the study. The rationale was that the study design was primarily aiming to test the feasibility of ozone treatment in an established model of sepsis [14]. Therefore, the use of such control animals would not affect the hypothesis testing of this feasibility study and hence not comply with the 3R of research animal ethics principle of reduction. Fourth, while the sepsis model was chosen because of our previous experience of this model, the 3.5-h time span was likely too short to detect significant improvement in sepsis parameters, which is why future studies should require longer observation times. Fifth, a control group was not used for the in vitro experiments. It is, therefore, possible that an effect of the system itself, without ozone, may confound the results, which should be investigated in future studies.

This feasibility study indicated that ozone may be useful for treatment of sepsis, independently of antibiotics, and may, therefore, be less susceptible to antibiotic resistance [5]. Other extracorporeal blood purification systems under development primarily aim to control the associated dysregulation of the immune system [36]. The ozonation system comprises a fundamentally new method of killing bacteria. The system may be considered in conjunction with hemodialysis, which requires catheterization of large veins. It is also possible that the system could be used with LPS adsorbers [37], for simultaneous antibacterial effects and removal of shock-inducing pathogens. Reduced bacteremia may still trigger an inflammatory response, and it is, therefore, possible that lowering bacterial levels should be combined with methods of lowering inflammation, to achieve the ultimate goal of lowering mortality in sepsis using methods other than antibiotics.

## Conclusions

Ozone decreased the concentration of viable *E. coli* in human whole blood. The system was technically feasible and physiologically tolerable in porcine sepsis/septic shock and should be considered for further studies towards clinical applications.

Take-home message: There are currently no methods other than antibiotics to kill bacteria in severe sepsis/septic shock. We here describe the feasibility of extracorporeal ozone administration, which shows potential to kill bacteria, and may be a new way forward in treating sepsis/septic shock.

## Data Availability

The data sets used and/or analyzed during the current study are available from the corresponding author on reasonable request.
